# Neddylation-mediated degradation of hnRNPA2B1 contributes to hypertriglyceridemia pancreatitis

**DOI:** 10.1038/s41419-022-05310-w

**Published:** 2022-10-11

**Authors:** Wei Chen, Yilong Wang, Wenwen Xia, Jinbao Zhang, Yan Zhao

**Affiliations:** 1 Department of Gastroenterology, Shanghai Tenth People’s Hospital of Tongji University, 200072 Shanghai, China; 2 Department of Critical Medicine, the First Hospital of Shanxi Medical University, 030000 Shanxi, China

**Keywords:** Cell death, Cell biology

## Abstract

Hypertriglyceridemia-induced acute pancreatitis (HTGP) is characterized by the acute and excessive release of FFA produced by pancreatic lipases. However, the underlying mechanisms of this disease remain poorly understood. In this study, we describe the involvement of the RNA binding protein hnRNPA2B1 in the development of HTGP. We used palmitic acid (PA) and AR42J cells to create a model of HTGP in vitro. RT-PCR and western blot analyses revealed a decrease in the level of hnRNPA2B1 protein but not mRNA expression in PA-treated cells. Further analyses revealed that hnRNPA2B1 expression was regulated at the post-translational level by neddylation. Restoration of hnRNPA2B1 expression using the neddylation inhibitor MLN4924 protected AR42J cells from PA-induced inflammatory injury by preventing NF-κB activation and restoring fatty acid oxidation and cell proliferation. Furthermore, RNA immunoprecipitation studies demonstrated that hnRNPA2B1 orchestrates fatty acid oxidation by regulating the expression of the mitochondrial trifunctional protein-α (MTPα). Administration of MLN4924 in vivo restored hnRNPA2B1 protein expression in the pancreas of hyperlipidemic mice and ameliorated HTGP-associated inflammation and pancreatic tissue injury. In conclusion, we show that hnRNPA2B1 has a central regulatory role in preventing HTGP-induced effects on cell metabolism and viability. Furthermore, our findings indicate that pharmacological inhibitors that target neddylation may provide therapeutic benefits to HTGP patients.

## Introduction

Acute pancreatitis (AP) is rapid inflammation of the pancreas that is characterized by the active release of pancreatic digestive enzymes into the pancreatic interstitium and systemic circulation, resulting in pancreatic autodigestion and systemic complications [1–3]. Common causes include gallstones, heavy alcohol consumption, and metabolic disorders [4]. Hypertriglyceridemia (HTG) is a well-documented metabolic cause of AP, accounting for 2–4% of all cases in the general population [5] and about 50% of cases during pregnancy [6]. Compared to AP of other etiologies, HTG-induced acute pancreatitis (HTGP) is associated with a higher risk of recurrent episodes and more severe complications such as pancreatic necrosis and renal dysfunction [6–9]. Current HTGP treatment involves a limited number of anti-hyperlipidemic modalities, including insulin and/or heparin that enhance lipoprotein lipase activity, apheresis that directly removes triglycerides (TG) from the circulation and oral anti-hyperlipidemic agents such as fibrates [10]. However, the efficacies of these anti-hyperlipidemic treatments are largely anecdotal, and need to be confirmed by large multicenter trials in the future. Understanding the cellular and molecular mechanisms that drive HTGP is critical for the development of novel, effective therapies for this undertreated disease.

Although the pathogenesis of HTGP is not fully understood, substantial evidence has pointed to the central role of lipotoxicity from FFA [11]. FFA are generated by the lipolysis of TG by lipases and are taken up by cells to be used as an energy source. FFAs are activated with ATP and coupled to coenzyme A (CoA) to generate acyl-CoA molecules. Acyl-CoA is transported into the mitochondrial matrix via the carnitine palmitoyltransferase (CPT1) transport pathway, where it undergoes β-oxidation to form acetyl-CoA, which enters the citric acid cycle [12]. Pancreatic lipases can hydrolyze excess TG to generate FFA, resulting in massive accumulation of FFA in the pancreas. FFA can also activate inflammatory cascades during the early stages of HTGP by inducing inflammatory cytokines such as TNF-α and IL-1β, and by inhibiting the anti-inflammatory cytokine IL-10 [13]. FFA can also trigger intracellular calcium release, leading to mitochondrial dysfunction, oxidative stress and ultimately, pancreatic necrosis [14].

The heterogeneous nuclear ribonucleoproteins (hnRNPs) are RNA binding proteins that complex with pre-mRNAs in the nucleus to influence pre-mRNA processing, metabolism, transport, and translation [15]. The *hnRNPA2B1* gene, which belongs to the A/B subfamily of hnRNPs, is involved in many physiological and pathophysiological processes through transcriptional regulation of a variety of target genes [16]. Of note, hnRNPA2B1 has been reported to ameliorate LPS-induced endothelial injury by downregulating NF-κB [17]. However, to our knowledge, the role of hnRNPA2B1 in HTGP pathogenesis has not been reported. In this study, we examined the levels of serum FFA and the severity of inflammation in HTGP patients. We subsequently investigated the role of hnRNPA2B1 in models of HTGP pathogenesis in in vitro and in vivo. Palmitic acid (PA) was used to induce an HTGP model in AR42J cells as described previously [18]. AR42J cells are currently the only cell line available that maintain many characteristics of normal pancreatic acinar cells in culture, such as synthesis and secretion of digestive enzymes, protein expression, and proliferation [19, 20]. Gonzalez. et. al showed that AR42J cell receptor expression and signaling mechanisms parallel those of pancreatic acinar cells. Therefore, this cell line has been widely used as an “in vitro” model to study the exocrine pancreas section [21]. PA induces acute pancreatic damage by increasing levels of palmitoleic acid ethyl ester, which can subsequently cause mitochondrial dysfunction in pancreatic cells [22]. In addition, we induced a model of HTGP in mice using poloxamer 407 (P-407) [23]. The administration of P-407 elevates serum triglyceride levels dose-dependently. Pancreatic injury is induced in HTG mice with cerulein, which has no effect on normal mice. Our findings demonstrate that neddylation-mediated degradation of hnRNPA2B1 contributes to the development of HTGP and suggests that specific neddylation inhibitors may be therapeutically beneficial to HTGP patients.

## Materials and methods

### Clinical specimens

Peripheral blood samples were collected from patients with confirmed HTGP who were admitted to the Department of Digestion of the Tenth People’s Hospital of Shanghai from 2017 to 2018. The diagnostic criteria for HTGP were serum triglycerides levels of ≥150 mg/dL under fasting conditions. Patients with AP with other inflammatory diseases were excluded from this study. A total of 64 blood samples were obtained from 7 normal healthy people and 57 HTGP patients. All patients provided written informed consent. The demographical characteristics of healthy subjects and patients are summarized in Table 1.

**Table 1:** The demographical characteristics of normal healthy people and patients.

	Patients		Normal healthy people	
	Male (%)	Female (%)	Male (%)	Female (%)
Sex	43 (67.19)	21 (32.81)	4 (57.14)	3 (42.86)
Age				
<60	38 (59.37)	14 (21.88)	3 (42.86)	2 (28.57)
>60	5 (7.81)	7 (10.94)	1 (14.29)	1 (14.29)

### Cell model of HTGP

The AR42J rat pancreatic cell line (CRL1492) obtained from ATCC was used for experiments as described previously [18, 24]. Cells were cultured in Dulbecco’s modified Eagle’s medium (Sigma-Aldrich, St. Louis, MO, USA) supplemented with 10% fetal bovine serum and 100 U/mL penicillin/streptomycin and incubated at 37 °C in a humidified atmosphere with 5% CO_2_. The HTGP model was induced by incubating AR42J cells with the indicated concentrations of PA.

### Animal model of HTGP

Eighteen C57BL/6 male mice weighing 20–25 g were purchased from Shanghai Model Organisms Center (Shanghai, China). Hyperlipidemia was induced by the intraperitoneal administration of P-407 (P2443, Sigma-Aldrich) in PBS at a dose of 500 mg/kg three times a week. On day 30, the hyperlipidemic mice received an intraperitoneal injection of cerulein (AnaSpec, Los Angeles, CA, USA) in PBS at a dose of 50 μg/kg every hour for a total of 10 injections. Then blood samples were collected from the tail vein and centrifuged for 15 min at 3,500 *g*, serum was obtained for further analysis.

To evaluate the effect of hnRNPA2B1 de-neddylation in HTGP mice, MLN4924 (30 mg/kg body weight) was administered subcutaneously, twice a week for two weeks. Animals were subsequently euthanized using intraperitoneal pentobarbital (100 mg/kg; P-010, Sigma-Aldrich).

### Cell transfection

The AR42J rat pancreatic acinar cell line (CRL1492™) was obtained from the ATCC and authenticated by the STR method and tested for mycoplasma contamination. For knockdown of mitochondrial trifunctional protein-α (MTPα), short hairpin RNAs (shRNAs) targeting MTPα (shMTPα) and a scramble shRNA (scr), which was used as the control, were synthesized by GenePharma (Shanghai, China). For MTPα knockdown studies, AR42J cells were transfected with pcDNA3.1-shMTPα or pcDNA3.1-scr plasmids using Lipofectamine™ 2000 (Invitrogen, Waltham, MA, USA) following the manufacturer’s instructions. For MTPα or hnRNPA2B1 overexpression studies, cells were transfected with pcDNA3.1-MTPα or pcDNA3.1-hnRNPA2B1 plasmids using Lipofectamine™ 2000. The transfection efficiency was determined by PCR and western blotting.

### Enzyme-linked immunosorbent assays

Serum amylase and lipase levels were used as markers of pancreatitis and were measured with an enzyme-linked immunosorbent assays (ELISA) Kit (ab137969, Abcam, Cambridge, UK; D799801, Sangon Biotech (Shanghai, China)). TNF-α, IL-1β and IL-10 levels in serum samples or tissue lysates were also determined using ELISA Kits (D711045, D711047, and D711114, respectively) from Sangon Biotech and iNOS enzymatic concentrations were evaluated using a kit from Abcam (ab253219, Abcam).

### Detection of serum FFA levels

Serum FFA levels were measured using a Free Fatty Acid Quantification Assay Kit obtained from Abcam (ab65341) following the manufacturer’s instructions.

### CPT1 activity

CPT1 activity was determined using a method reported previously [25, 26]. Briefly, 10 μl cells (5 × 10^5^) or tissue homogenate was transferred into a microcentrifuge tube and preincubated for 10 min at 30 °C. The reaction was initiated by adding 90 μl reaction mixture to the microcentrifuge tube. The reaction was terminated by the addition of 60 μl 1.2 mM ice-cold HCl. The generated [^3^ H]-palmitoylcarnitine was extracted with water-saturated butanol and levels were quantified using a liquid scintillation counter.

### Fatty acid oxidation

The rate of fatty acid oxidation in AR42J cells or tissue lysate was evaluated using a Fatty Acid Oxidation Complete Assay Kit from Abcam (ab222944) following the manufacturer’s instructions.

### Cell viability

Cell viability was determined using a CCK8 assay (ab228554, Abcam) following the manufacturer’s instructions. Briefly, cells were incubated with 10 μL CCK-8 solution for 2 h. The absorbance at 490 nm was recorded on a spectrophotometer.

### Quantitative real-time polymerase chain reaction

Total RNA was extracted from cells or tissue homogenate using Trizol (Invitrogen) and reverse transcribed using PrimeScript RT Master Mix (RR036A, Takara, Kusatsu, Japan). The mRNA levels were determined on a 7300 real-time PCR system (Applied Biosystems, Waltham, MA, USA) using SYBR green (4444432, Applied Biosystems). The relative expression values were calculated using the 2^−∆∆Ct^ method. GAPDH served as the internal control. All experiments were performed in triplicate. The primers used are listed in Supplementary Table S1.

### Western blot analysis

Whole cell extracts were prepared in RIPA buffer containing a protease inhibitor cocktail. Total protein was separated by SDS-PAGE. The following antibodies were used: anti-hnRNPA2B1 (ab31645, Abcam, 1:500), anti-NEDD8 (ab81264, Abcam, 1:5000), anti-NF-κB1 (ab209795, Abcam, 1:1000), anti-TRAF2 (ab126758, Abcam, 1:1000), anti-MTPα (PA5-29813, Invitrogen, 1:1500), and anti-actin (ab8226, Abcam, 1:500).

### Co-immunoprecipitation

Cells were harvested and lysed in RIPA buffer containing a protease inhibitor cocktail. Protein G beads (Dynabeads, Thermo Fisher Scientific, Waltham, MA, USA) were incubated with primary antibodies for 10 min and then overnight at 4 °C. The protein lysates (1–2 mg each) were incubated with RNase A and control isotype antibody for 30 min at 4 °C. The beads and primary antibody-bound beads were incubated with precleared lysates overnight at 4 °C. The precipitated proteins were eluted in NuPAGE LDS sample buffer, heated at 70 °C for 10 min, and immunoblotted with antibodies against hnRNPA2B1 and NEDD8.

### RNA immunoprecipitation

RNA immunoprecipitation (RIP) assays were carried out using an Imprint RNA Immunoprecipitation Kit from Sigma-Aldrich. For hnRNPA2B1 pull-down, MTPα mRNA was synthesized using T7 RNA polymerase (18033019, Invitrogen), and the purified product was labeled with biotin. The cell lysate was incubated with biotin-labeled MTPα mRNA or biotin-labeled antisense MTPα ΗΝ (as negative control), followed by incubation with streptavidin-coated beads. The co-precipitated proteins were detected by western blot analysis. The cell lysate served as the input. For MTPα mRNA pull-down, the cell lysate was incubated with beads pre-coated with anti-hnRNPA2B1 antibody or IgG (as negative control). The co-precipitated RNAs were isolated and analyzed by quantitative real-time polymerase chain reaction (qRT-PCR).

### Immunofluorescence

Formalin-fixed, paraffin-embedded mouse pancreatic sections were dewaxed, rehydrated, and incubated with blocking buffer containing 1% BSA and 5% normal goat serum for 1 h. Then, samples were incubated with antibodies against caspase-1 (MA1-83977, Invitrogen), NF-κB1 (ab209795, Abcam), MTPα (PA5-29813, Invitrogen), hnRNPA2B1 (ab31645, Abcam), and Ki67 (ab15580, Abcam, 1:200) in blocking buffer overnight at 4 °C. After washing with PBS, slides were incubated with Alexa Fluor 488-conjugated anti-rabbit IgG (Invitrogen, 1:500) or Alexa Fluor 555-conjugated anti-mouse IgG (Invitrogen, 1:500) and subjected to immunofluorescence imaging analysis on confocal laser scanning microscope.

### Hematoxylin and eosin staining

Mouse pancreatic tissues were fixed in formalin for 24 h, washed in 70% ethanol, and embedded in paraffin. The sections (0.5 cm × 0.5 cm specimen) were cut, dewaxed and stained with Hematoxylin and eosin (H&E). Images were analyzed using Aperio ImageScope software (Leica, Wetzlar, Germany).

### Flow cytometry analysis of apoptosis

Treated cells were digested with 0.25% trypsin, centrifuged at 1,500 g for 5 min and resuspended. The assay was performed using an AnnexinV Apoptosis Detection Kit (E-CK-A218, Wuhan, China). Briefly, cells were resuspended in 50 μL of binding buffer containing 5 μL 7-AAD dye and incubated for 15 min in the dark. Following the addition of 450 μL the binding buffer and 1 μL Annexin V-APC, the reaction mix was incubated for 15 min. Levels of apoptosis were determined using a flow cytometer (Beckman Coulter, Brea, CA, USA).

### Statistical analysis

All data are presented as mean ± SD. Data were analyzed with SPSS 13.0. The Tukey range test and *t* test were used to evaluate the differences among more than three groups and between two groups, respectively. *P* < 0.05 was deemed statistically significant.

## Results

### Lipid and cytokine profiles of HTGP patients and HTGP mice

**Fig. 1:**
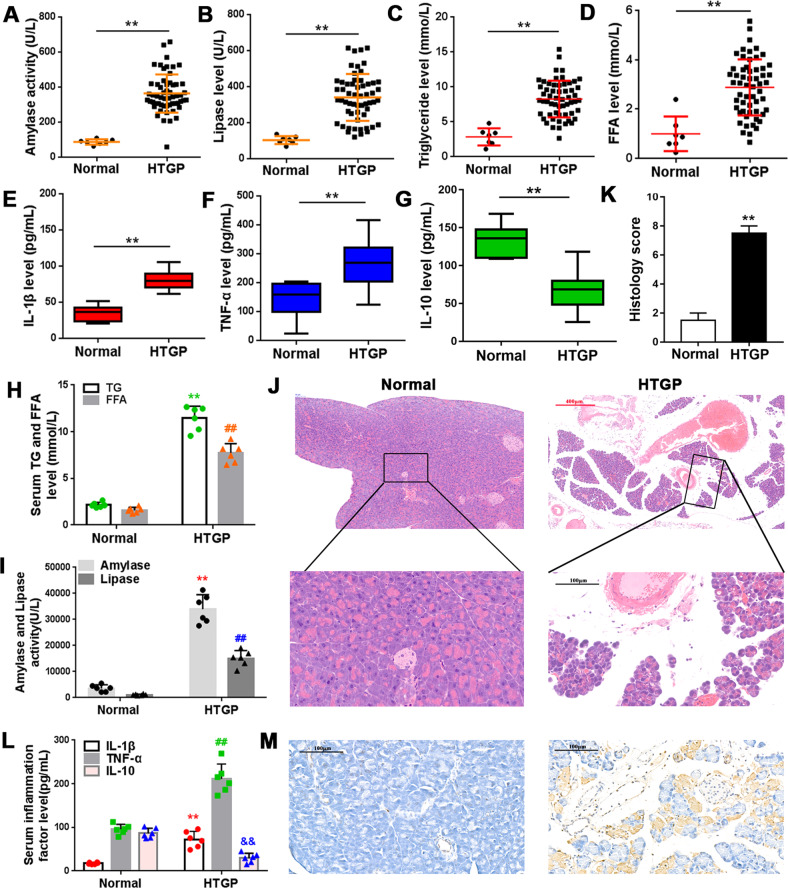
Lipid and cytokine profiles of HTGP patients and HTGP mice. (**A**–**D**) Amylase (**A**), lipase (**B**), TG (**C**) and FFA (**D**) levels were detected in the sera of HTGP patients by ELISA. n = 64 (Normal = 7, HTGP = 57), *P < 0.05, **P < 0.01, ***P < 0.001. **E**–**G** The inflammatory response was determined by measuring IL-1β (**E**), TNF-α (**F**), and IL-10 (**G**) levels in patient sera. **H**, **I**, **L** Amylase (**H**), lipase (**I**) and inflammatory cytokine (**L**) levels were detected in control and HTGP-induced mice by ELISA. n = 6; ^&^*P* < 0.05, **^, ##^*P* < 0.01 vs. Normal. **J** H&E and Histology score (**K**) and (**M**) TUNEL staining of the pancreatic tissue of normal and HTGP mice. Scale bar = 100 μm.

HTGP patients exhibited elevated amylase and lipase activities (Fig. 1A, B), and increased serum TG and FFA levels (Fig. 1C, D) compared to control subjects. We also observed an increase in IL-1β and TNF-α levels (Fig. 1E, F). In contrast, IL-10 levels showed a negative correlation (Fig. 1G). Next, we administered P-407 and cerulein to induce hyperlipidemia in mice. Consistent with the clinical data, HTGP mice exhibited higher serum TG and FFA levels and elevated amylase and lipase activities than the controls (Fig. 1H, I). H&E (Fig. 1J, K) and TUNEL staining (Fig. 1M) revealed typical histopathological features of AP including interlobular edema, inflammatory cell infiltration and acinar cell apoptosis in the HTGP pancreas compared to normal tissue. In addition, significantly increased IL-1β and TNF-α levels and reduced IL-10 levels were observed in HTGP mice (Fig. 1L).

### PA induces an inflammatory response in AR42J cells in vitro

**Fig. 2:**
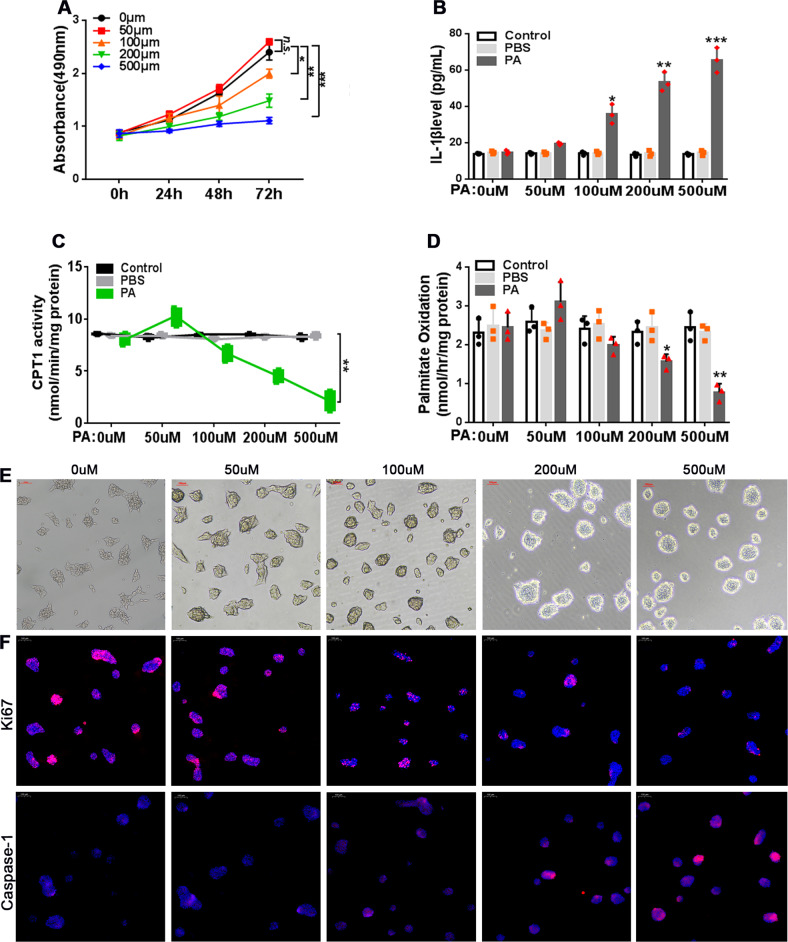
PA induces an inflammatory response in AR42J cells in vitro. **A** AR42J cells were treated with PA at the indicated concentrations for up to 72 h. Cell viability was determined using the CCK-8 assay. **B**–**F** AR42J cells were treated with PA at the indicated concentrations for 12 h. **B** IL-1β levels were measured in the culture supernatant by ELISA. **C** CPT1 enzymatic activity was assessed. **D** The rate of fatty acid oxidation was determined by analyzing palmitate oxidation levels. *n* = 3; *P < 0.05, **P < 0.01, ***P < 0.001 (or vs. 0 μM *P*A); n.s. = no statistically significant differences. **E** Phase contrast microscopy was used to visualize morphological changes in the cells. **F** Representative immunofluorescence images showing Ki67 (upper panel) and caspase-1 (lower panel) staining. Red, Ki67, or caspase-1; blue, DAPI, scale bar = 100 μm.

To test the effects of exogenous FFA, we incubated AR42J rat pancreatic cells with 0–500 μM PA. PA inhibited cell proliferation in a dose-dependent manner (Fig. 2A). Furthermore, cells treated with PA for 12 h exhibited increased IL-1β secretion (Fig. 2B), decreased CPT1 enzymatic activity (Fig. 2C), and impaired fatty acid oxidation (Fig. 2D), representative of an inflammatory response and mitochondrial dysfunction. In addition, we observed changes in cell morphology (shrinkage to roundness) that were specific to PA-treated AR42J cells (Fig. 2E). Ki67 and caspase-1 staining revealed decreased proliferative activity and increased apoptosis in PA-treated AR42J cells compared to the controls (Fig. 2F).

### PA promotes neddylation-mediated hnRNPA2B1 protein degradation in AR42J cells

**Fig. 3:**
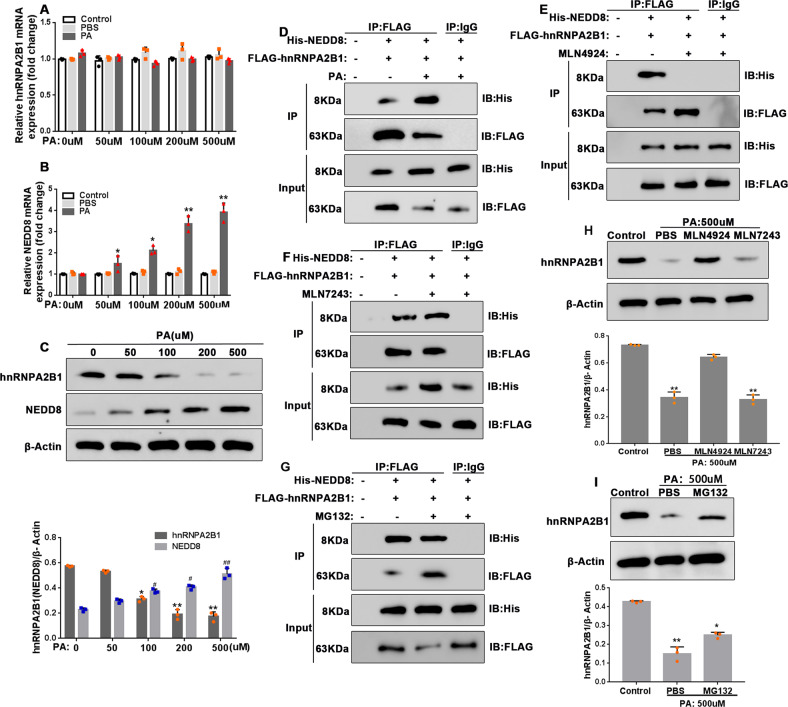
PA promotes neddylation-mediated hnRNPA2B1 protein degradation. **A**–**C** AR42J cells were treated with PA at the indicated concentrations for 12 h. **A**, **B** Relative mRNA expression levels of hnRNPA2B1 (**A**) and NEDD8 (**B**) were assessed by qRT-PCR. **C** hnRNPA2B1 and NEDD8 protein expression levels were examined by western blot analysis. *n* = 3; *P < 0.05, **P < 0.01 vs^.^ 0 μM PA. **D**–**F**, **H** AR42J cells co-transfected with His-NEDD8 and Flag-hnRNPA2B1 were treated with 500 μM PA for 12 h without (**D**) or with 2 h pre-treatment with 3 mM MLN4924 (a neddylation inhibitor) (**E**), 1 mM MLN7243 (a ubiquitylation inhibitor) (**F**), or 10 μM MG132 (a proteasome inhibitor) (**G**). Cell lysates were immunoprecipitated with anti-FLAG antibody, then immunoblotted with antibodies against FLAG and His. The cell lysates (Input) served as the control. **H**, **I** AR42J cells were pre-treated with 3 mM MLN4924 or 1 mM MLN7243 (**H**), or 10 μM MG1342 (**I**) for 2 h, and subsequently treated with 500 μM PA for 12 h. hnRNPA2B1 protein levels were examined by western blot analysis.

To determine whether hnRNPA2B1 has a role in PA cytotoxicity, we evaluated hnRNPA2B1 mRNA and protein expression in AR42J cells. Unexpectedly, PA treatment led to a reduction in hnRNPA2B1 protein levels, while mRNA expression was unaffected (Fig. 3A–C), suggesting that post-transcriptional regulation was responsible for the reduction in protein levels. Interestingly, we found that the decrease in hnRNPA2B1 protein expression was accompanied by increased expression of the ubiquitin-like protein NEDD8 (Fig. 3B, C).

Co-immunoprecipitation experiments confirmed that hnRNPA2B1 interacted directly with NEDD8 (Fig. 3D, full and uncropped western blots are in Supplemental Material). Furthermore, we showed that the hnRNPA2B1-NEDD8 interaction was disrupted by MLN4924 pre-treatment (Fig. 3E, full and uncropped western blots are in Supplemental Material), but not by pre-treatment with MLN7243 (Fig. 3F) or MG132 (Fig. 3G, full and uncropped western blots are in Supplemental Material). To determine whether neddylation or ubiquitination was involved in the regulation of hnRNPA2B1 by PA, cells were pre-treated with the neddylation inhibitor MLN4924, the ubiquitylation inhibitor MLN7243, or the proteasome inhibitor MG132 before PA treatment. Western blot analysis revealed that MLN4924 and MG132 treatment, but not MLN7243 treatment, restored hnRNPA2B1 protein expression in PA-treated cells (Fig. 3H, I, full and uncropped western blots are in Supplemental Material), suggesting that PA-induced neddylation, and not ubiquitylation-dependent proteasomal degradation, was responsible for the PA-dependent reduction in hnRNPA2B1 protein expression. Taken together, our findings demonstrate that PA downregulated hnRNPA2B1 expression by inducing neddylation-mediated proteasomal degradation of the protein.

### HnRNPA2B1 overexpression inhibits PA-induced NF-κB activation and inflammation

**Fig. 4:**
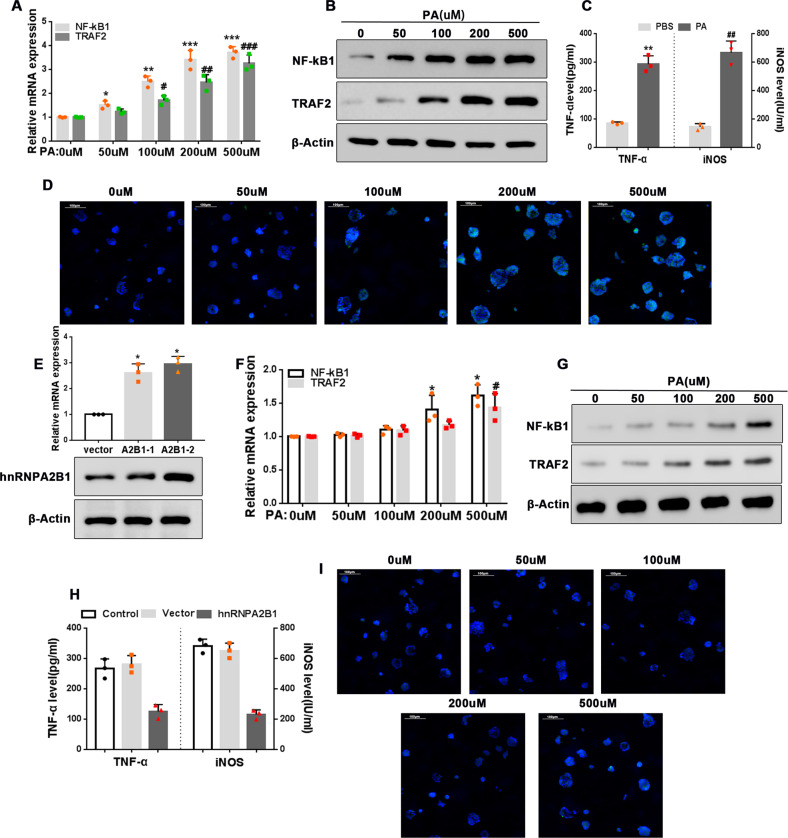
hnRNPA2B1 overexpression inhibits NF-κB activation by PA. **A**, **B**, **D** AR42J cells were treated with PA at the indicated concentrations for 12 h. **A** Relative mRNA expression levels of NF-κB1 and TRAF2 were assessed by qRT-PCR. **B** NF-κB1 and TRAF2 protein expression levels were examined by western blot analysis. **D** Representative immunofluorescence images showing staining for the NF-κB subunit P65. Green, P65; blue, DAPI, scale bar = 100 μm. *n* = 3; *, ^#^*P* < 0.05, **^, ##^*P* < 0.01, ***^, ###^*P* < 0.001 vs. 0 μM PA. **C** AR42J cells were treated with 500 μM PA for 12 h. TNF-α levels and intracellular iNOS concentrations were measured by ELISA. *n* = 3; *P < 0.05, ^##^*P* < 0.01 vs. PBS. **E** AR42J cells were transfected with the pcDNA3.1-hnRNPA2B1 plasmid or empty vector. hnRNPA2B1 mRNA and protein levels were assessed by qRT-PCR and western blot analyses, respectively. *n* = 3, *P < 0.05 vs. vector. **F**, **G**, **I** AR42J cells transfected with the pcDNA3.1-hnRNPA2B1 plasmid were treated with PA at the indicated concentrations for 12 h. **F** Relative mRNA expression levels of NF-κB1 and TRAF2 were determined by qRT-PCR. **G** NF-κB1 and TRAF2 protein levels were examined by western blot analysis. **I** Representative immunofluorescence images showing staining for P65. Green, P65; blue, DAPI, scale bar = 100 μm. *n* = 3; *^, #^*P* < 0.05, **P < 0.01 vs. 0 μM PA. **H** AR42J cells transfected with the pcDNA3.1-hnRNPA2B1 plasmid or empty vector were treated with 500 μM PA for 12 h. TNF-α levels and intracellular iNOS concentrations were determined by ELISA. n = 3, *^, #^*P* < 0.05 vs. vector.

To further investigate PA-induced inflammation, we examined the NF-κB pathway in AR42J cells. Treatment with PA increased the mRNA and protein expression levels of NF-κB1 and its upstream activator TRAF2 (Fig. 4A, B, full and uncropped western blots are in Supplemental Material). In addition, PA treatment led to an increase in TNF-α secretion and intracellular iNOS concentrations (Fig. 4C), suggesting that an NF-κB-mediated inflammatory response had been activated. PA treatment also increased the expression of the NF-κB subunit P65 (Fig. 4D). To examine the effects of hnRNPA2B1 overexpression, we transfected AR42J cells with the pcDNA3.1-hnRNPA2B1 plasmid. Overexpression of hnRNPA2B1 was confirmed by both qRT-PCR and western blot analysis (Fig. 4E, full and uncropped western blots are in Supplemental Material). We found that hnRNPA2B1 overexpression inhibited PA-induced activation of the NF-κB signaling pathway (Fig. 4F–I, full and uncropped western blots are in Supplemental Material). Taken together, our results indicate that PA induces activation of the NF-κB pathway via neddylation of hnRNPA2B1.

### MTPα expression is post-transcriptionally regulated by hnRNPA2B1 in PA-treated AR42J cells

**Fig. 5:**
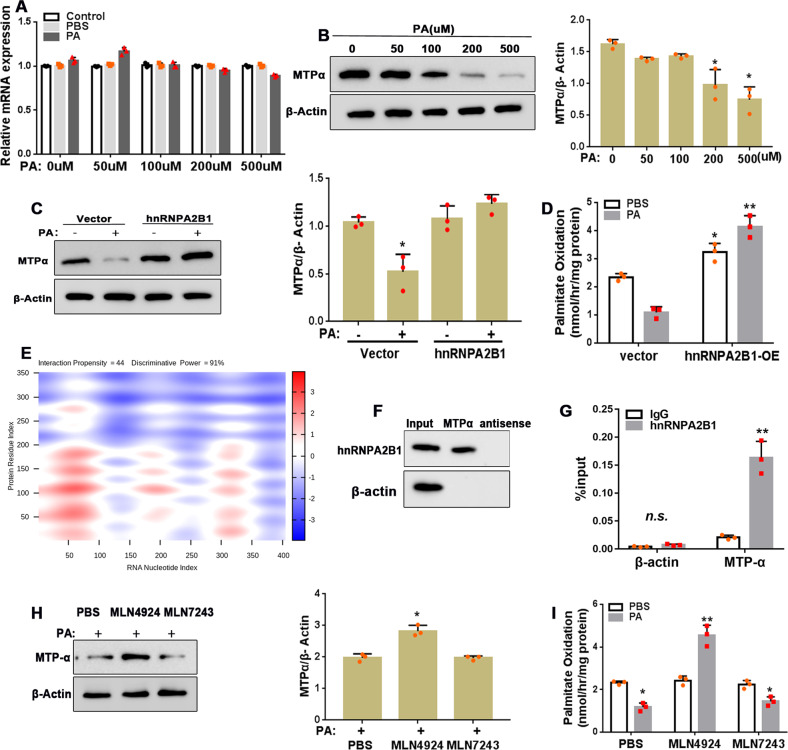
hnRNPA2B1 binds to MTPα mRNA and its overexpression restores MTPα protein levels downregulated by PA. **A**, **B** AR42J cells were treated with PA at the indicated concentrations for 12 h. MTPα mRNA (**A**) and protein (**B**) levels were determined by qRT-PCR and western blot analyses, respectively. *n* = 3. AR42J cells transfected with the pcDNA3.1-hnRNPA2B1 plasmid or empty vector were treated with 500 μM PA for 12 h. MTPα protein levels (**C**) and fatty acid oxidation rate (**D**) were determined by western blot analysis and ELISA, respectively. **E** *CatRAPI* prediction of the hnRNPA2B1 protein and MTPα mRNA interaction propensity (http://service.tartaglialab.com/page/catrapid group). **F**, **G** RNA pull-down and RIP assays were used to confirm the hnRNPA2B1 protein/MTPα mRNA interaction in AR42J cells. *n* = 3, **P < 0.01 vs. IgG. AR42J cells were treated with 500 μM PA for 12 h with or without pre-treatment with 3 mM MLN4924 or 1 mM MLN7243 for 2 h. MTPα protein levels (**H**) and fatty acid oxidation rate (**I**) were measured by western blot analysis and ELISA, respectively. *n* = 3; *P < 0.05, **P < 0.01 vs. PBS.

To further investigate PA-induced impairment in fatty acid oxidation, we evaluated the mRNA and protein expression levels of MTPα, a key enzyme that catalyzes multiple steps in β oxidation. PA treatment had no effect on MTPα mRNA expression (Fig. 5A), but reduced MTPα protein level in a dose-dependent manner (Fig. 5B, full and uncropped western blots are in Supplemental Material). HnRNPA2B1 overexpression restored MTPα protein expression that was otherwise downregulated by PA (Fig. 5C, full and uncropped western blots are in Supplemental Material) and increased the rate of fatty acid oxidation (Fig. 5D). To explore the underlying mechanism, we used the *CatRAPI* algorithm to estimate the binding propensity between hnRNPA2B1 protein and MTPα mRNA and obtained a high score of 44 (Fig. 5E). RNA pull-down and RIP experiments confirmed direct binding between hnRNPA2B1 protein and MTPα mRNA (Fig. 5F, G, full and uncropped western blots are in Supplemental Material), suggesting that hnRNPA2B1 upregulated MTPα protein expression by binding to its mRNA and promoting translation. Moreover, MLN4924 but not MLN7243 pre-treatment restored MTPα protein and fatty acid oxidation downregulated by PA (Fig. 5H, I, full and uncropped western blots are in Supplemental Material). These restorative effects of MLN4924 were presumably mediated through inhibition of neddylation and subsequent restoration of hnRNPA2B1 protein levels that were downregulated by PA.

### HnRNPA2B1 overexpression restores fatty acid oxidation and cell proliferation through MTPα

**Fig. 6:**
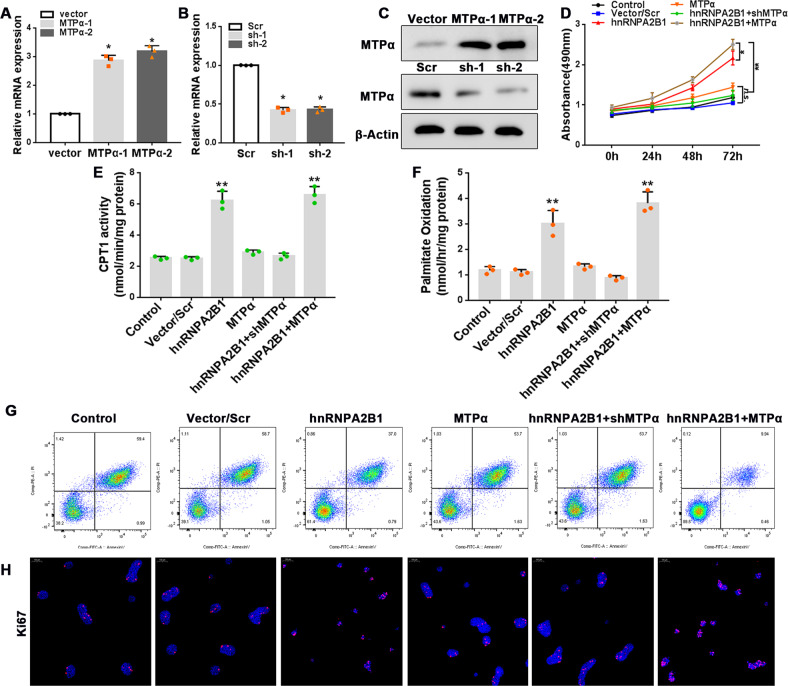
hnRNPA2B1 promotes fatty acid oxidation and cell proliferation by increasing MTPα expression. **A**–**C** AR42J cells were transfected with the pcDNA3.1-MTPα, pcDNA3.1-shMTPα, or pcDNA3.1-scr plasmid or the empty vector. MTPα mRNA (**A**, **B**) and protein (**C**) levels were determined by qRT-PCR and western blot analyses, respectively. n = 3; *P < 0.05 vs. vector or scr. **D**–**H** AR42J cells were transfected with the pcDNA3.1-scr, pcDNA3.1-MTPα, or pcDNA3.1-hnRNPA2B1 plasmid or co-transfected with the pcDNA3.1-hnRNPA2B1 and pcDNA3.1-MTPα or pcDNA3.1-hnRNPA2B1 and pcDNA3.1-shMTPα plasmids for up to 72 h in the presence of 500 μM PA. **D** Cell viability was determined using the CCK-8 assay. **E** CPT1 enzymatic activity was evaluated. **F** The rate of fatty acid oxidation was assessed. **G** Levels of apoptosis were measured by flow cytometry. **H** Representative immunofluorescence images showing Ki67 staining. Red, Ki67; blue, DAPI; scale bar = 100 μm. *n* = 3, **P < 0.01 vs. vector/scr, n.s. = no statistically significant differences.

To determine how the interactions between hnRNPA2B1 and MTPα regulate fatty acid oxidation and cell proliferation, we studied the effects of MTPα or hnRNPA2B1 overexpression alone or combined with a combination of hnRNPA2B1 overexpression and MTPα knockdown. MTPα overexpression and knockdown were confirmed by both qRT-PCR and western blotting (Fig. 6A–C, full and uncropped western blots are in Supplemental Material). Although MTPα overexpression alone showed no significant effects, hnRNPA2B1 overexpression restored CPT1 activity, fatty acid oxidation, and cell proliferation that was downregulated by PA (Fig. 6D–H). Of note, the restorative effects of hnRNPA2B1 overexpression were enhanced by MTPα overexpression but inhibited by MTPα knockdown (Fig. 6D–H), suggesting that the effects of hnRNPA2B1 were mediated by the upregulation of MTPα.

### MLN4924 protects AR42J cells from PA-induced inflammatory injury

**Fig. 7:**
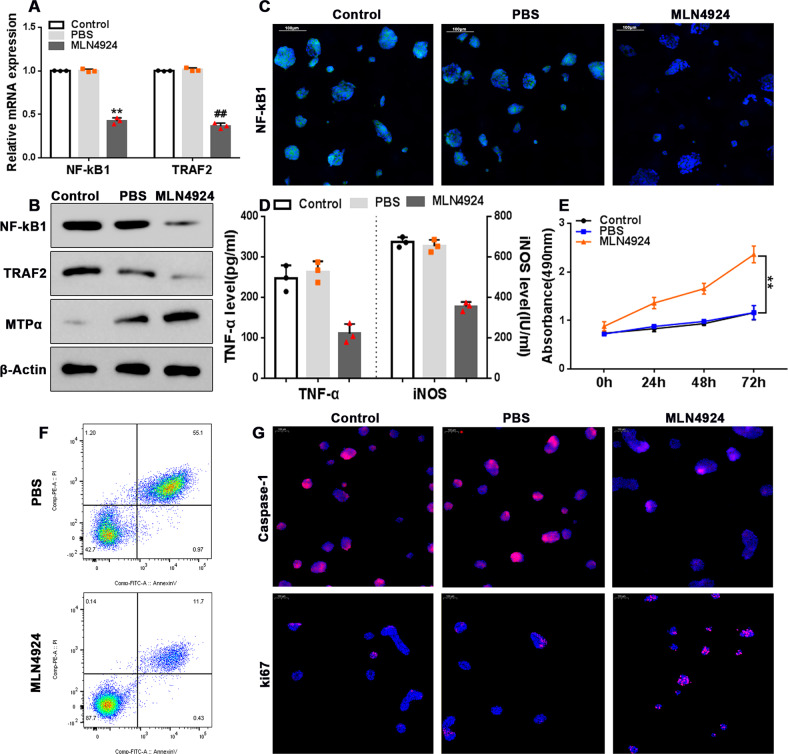
MLN4924 protects AR42J cells from PA-induced inflammatory injury. **A**–**D**, **F**, **G** AR42J cells were treated with 500 μM PA for 12 h with or without pre-treatment with 3 mM MLN4924 for 2 h. **A** NF-κB1 and TRAF2 mRNA levels were determined by qRT-PCR. **B** NF-κB1, TRAF2, and MTPα protein levels were assessed by western blot analysis. **C** Representative immunofluorescence images showing P65 staining. Red, P65; blue, DAPI; scale bar = 100 μm. **D** TNF-α levels and intracellular iNOS concentrations were determined by ELISA. **F** Flow cytometry was used to determine the level of apoptosis. **G** Representative immunofluorescence images showing caspase-1 and TUNEL staining. Red, caspase-1 or TUNEL; blue, DAPI; scale bar = 100 μm. **E** AR42J cells were treated with PA at the indicated concentrations for up to 72 h with or without pre-treatment with 3 mM MLN4924 for 2 h. Cell viability was determined using the CCK-8 assay. *n* = 3; *^, #^*P* < 0.05, **^, ##^*P* < 0.01 vs. PBS; n.s. = no statistically significant differences.

Having established that hnRNPA2B1 overexpression protected AR42J cells from PA-induced inflammation and cytotoxicity, we speculated that neddylation inhibitors would exhibit similar protective effects by blocking neddylation-mediated hnRNPA2B1 degradation. Indeed, treating AR42J cells with the neddylation inhibitor MLN4924 before PA treatment prevented NF-κB activation (Fig. 7A–D), reduced apoptosis (Fig. 7F, G), and restored MTP-α expression and cell proliferation (Fig. 7B, E, full and uncropped western blots are in Supplemental Material).

### Administration of MLN4924 ameliorates HTGP in mice

**Fig. 8:**
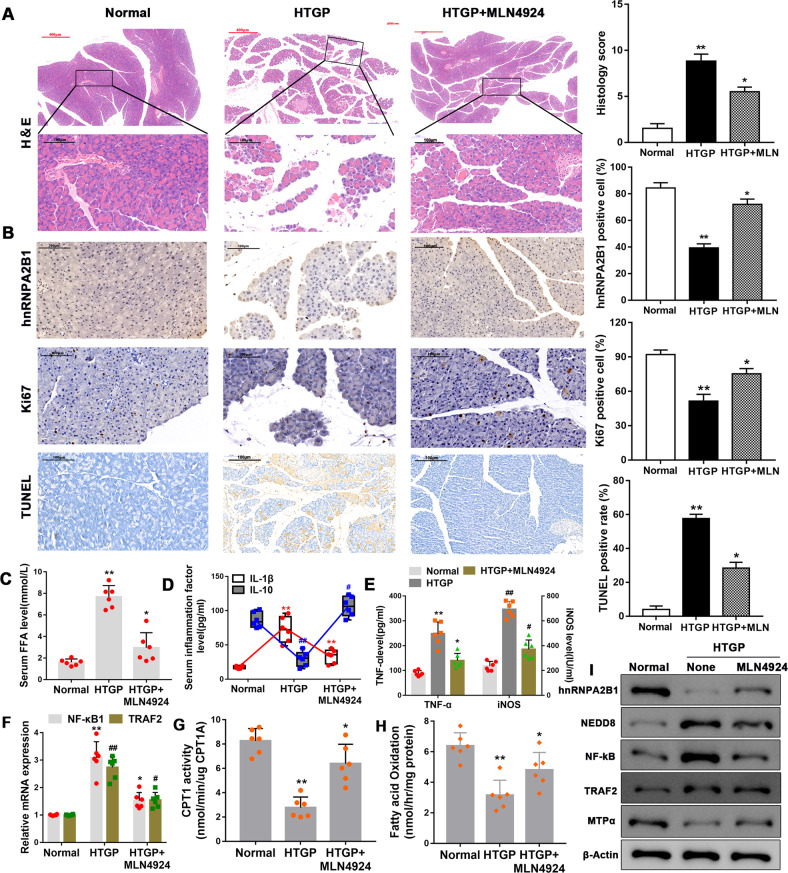
Administration of MLN4924 ameliorates HTGP in mice. **A** Representative images and histology score analyses showing H&E staining of the pancreatic tissue. **B** Representative images and quantitative analyses showing immunohistochemical staining of hnRNPA2B1 (upper panel) and Ki67 (middle panel), and TUNEL (lower panel) staining of the pancreatic tissue. Scale bar = 100 μm. **C**–**E** FFA (**C**), IL-1β and IL-10 (**D**), and TNFα (**E**) serum levels and iNOS concentrations in the pancreatic tissue (**E**) were determined by ELISA. **F** Relative NF-κB and TRAF2 mRNA levels were evaluated in the pancreatic tissue by qRT-PCR. **P < 0.01,*P < 0.05, ^##^*P* < 0.01, ^#^*P* < 0.05. **G** C*P*T1 activity was measured in the pancreatic tissue. **H** The rate of fatty acid oxidation was determined in the pancreatic tissue. **I** hnRNPA2B1, NEDD8, NF-κB, TRAF2, and MTPα protein levels were assessed in the pancreatic tissue by western blot analysis.

To determine whether our in vitro findings could be extrapolated to in vivo models of HTGP, we established a mouse model of HTGP by administering P-407 and cerulein to C57BL/6 mice. H&E, TUNEL, and Ki67 staining confirmed histopathological features of AP (Fig. 8A, B). We found that HTGP mice exhibited higher serum FFA (Fig. 8C), IL-1β (Fig. 8D) and TNFα (Fig. 8E), lower IL-10 levels (Fig. 8D), and higher NF-κB (Fig. 8F), TRAF2 expression (Fig. 8F) and iNOS concentrations (Fig. 8E) in the pancreas compared to control mice, indicating that HTGP led to systemic and pancreatic inflammation. The pancreas of HTGP-induced mice showed lower CPT1 activity (Fig. 8G) and MTPα protein expression (Fig. 8I, full and uncropped western blots are in Supplemental Material). Of note, these deleterious changes in the pancreas of HTGP-treated mice were accompanied by an increase in NEDD8 and decrease in hnRNPA2B1 protein levels compared to control mice (Fig. 8I). Administration of the neddylation inhibitor MLN4924 restored pancreatic hnRNPA2B1 expression, inhibited systemic and pancreatic inflammation, and prevented mitochondrial dysfunction and histopathological changes in the pancreas of HTGP-treated mice (Fig. 8A–H). Thus, our in vivo data, together with our in vitro findings, strongly suggest that FFA-induced, neddylation-mediated degradation of the hnRNPA2B1 protein plays a critical role in the pathogenesis of HTGP.

## Discussion

Improvements in early diagnosis and timely intervention have led to a significant decrease in AP mortality over the past decade from 1.6% to 0.8% [27]. However, the worldwide incidence of AP has been increasing, along with a rising incidence of AP-associated short- and long-term complications [28]. These upward trends are likely driven by the climbing global obesity epidemic [29, 30], making HTGP an increasingly prominent disease that demands effective treatment. In the present study, we detected the levels of serum FFA and the severity of inflammation in HTGP patients. We subsequently demonstrated that PA activated NF-κB and induced inflammatory injury in AR42J cells in vitro. Our mechanistic studies identified neddylation-mediated degradation of the RNA binding protein hnRNPA2B1 as a central mechanism responsible for these in vitro detrimental effects of FFA, and contributed to the inflammatory damage of the pancreas in HTGP mice. These findings create more evidence on the molecular pathophysiology of HTGP, and may be beneficial in the development of novel treatment strategies for this disease.

In this study, HTGP was induced in mice by the administration of P-407 and cerulein. Compared to control mice, HTGP-induced mice exhibited typical pathophysiological features of HTGP including elevated serum TG/FFA levels and inflammatory injury of the pancreas manifested as interlobular edema, inflammatory cell infiltration, and increased acinar cell apoptosis. Of note, these pathological changes were accompanied by increased pancreatic NF-κB activation and systemic inflammation. The NF-κB pathway plays a key role in AP pathogenesis by initiating the inflammatory cascade in the pancreas during its early stages [31, 32]. Moreover, studies have shown that excess lipids can exacerbate AP by enhancing NF-κB activation. For example, in a rat model of taurocholate-induced AP, a high fat diet aggravated NF-κB activation in the pancreas and enhanced pathological tissue damage [33]. In ex vivo experiments with isolated rat pancreatic cells, stimulation with FFA triggered an NF-κB-mediated inflammatory response [34]. Here, we found that treating cultured pancreatic cells with PA led to the activation of NF-κB and its downstream targets TNF-α, IL-1β, and iNOS. Our in vitro findings, together with previous in vivo and ex vivo results [33, 34], demonstrated that activation of the NF-κB pathway is a key mechanism driving HTGP. Furthermore, the PA-treated pancreatic cells, as well as the pancreas of HTGP-induced mice, exhibited a decreased rate of fatty acid oxidation compared to their corresponding controls. Thus, in addition to inflammation, FFA-induced mitochondrial dysfunction may also contribute to pancreatic tissue injury in HTGP. This is in agreement with the findings that PA can inhibit cytosolic and mitochondrial ATP levels in pancreatic cells and generate toxic cytosolic Ca^2+^ signals [35]. In fact, the deleterious effects of PA can be counteracted by directly supplying ATP to the cytosol [3, 36].

HnRNPA2B1 has previously been shown to have an anti-inflammatory role in autoimmune endocrine disorders [37]. In addition, hnRNPA2B1 has been shown to protect vascular endothelial cells from LPS-induced inflammatory injury by downregulating NF-κB [17]. Consistent with these studies, we found that the overexpression of hnRNPA2B1 in pancreatic cells prevented PA-induced NF-κB activation. Neddylation-mediated degradation of key immunoregulatory molecules appears to play a critical role in the development of immune-related diseases [38]. In this study, we found that PA upregulated NEDD8 expression and stimulated neddylation-mediated degradation of hnRNPA2B1, causing a decrease in the hnRNPA2B1 protein levels with no effect on mRNA levels. This reduction in hnRNPA2B1 protein levels resulted in NF-κB activation in PA-treated pancreatic cells. Co-immunoprecipitation assays confirmed direct interactions between NEDD8 and hnRNPA2B1. Furthermore, using specific neddylation or ubiquitination inhibitors, we demonstrated that hnRNPA2B1 degradation was mediated by neddylation and not ubiquitination of the protein. Of note, hnRNPA2B1 overexpression also upregulated MTPα and restored the rate of fatty acid oxidation in pancreatic cells downregulated by PA. RIP assays revealed a direct association between hnRNPA2B1 protein and MTPα mRNA, providing a possible mechanism for this regulation. Thus, hnRNPA2B1 appears to protect pancreatic cells against lipotoxicity by simultaneously suppressing inflammation and boosting mitochondrial function. Furthermore, administration of the neddylation inhibitor MLN4924 restored hnRNPA2B1 protein levels in the pancreas of HTGP-induced mice in vivo and ameliorated HTGP-associated inflammation and pancreatic mitochondrial dysfunction and tissue injury.

There are some limitations to our study. Sub-type differences in AR42J pancreatic cells may influence the results. For instance, some AR42J cells lack cholecystokinin receptors and may have significant neuronal phenotypes, unlike pancreatic acinar cells [24]. Although the results from AR42J cells can closely resemble those found in rodent pancreatic acinar cell lines, to address the variability between pancreatic cell lines and to strengthen the evidence, we also conducted our experiments in an animal model of HTGP.

Future studies will focus on determining the clinical relevance of hnRNPA2B1 and neddylation inhibitors in HTGP. The expression of hnRNPA2B1 has been implicated in various cancers, and its clinical use as a potential biomarker for cancer has been discussed [39, 40]. In addition, the targeting of RBPs has also emerged as a critical therapeutic strategy for the treatment of ALS [41]. However, to date, the relevance of hnRNPA2B1 as a potential therapeutic target has not been tested in clinical trials. In contrast, several clinical trials examining the therapeutic potential of neddylation inhibitors against cancer [42, 43], as well as in neurological diseases [44] are in progress. Future studies will help determine whether neddylation inhibitors may be a beneficial therapeutic approach for HTGP, potentially through the regulation of hnRNPA2B1.

## Supplementary information

Supplementary Table S1

aj-checklist

Fig.3-raw blots

Fig.4-raw blots

Fig.(5+6+7)-raw blots

Fig.8-raw blots

## Data Availability

The raw data supporting the conclusions of this article will be made available by the authors upon reasonable request.
