# Supplementary material for: Neddylation-mediated degradation of hnRNPA2B1 contributes to hypertriglyceridemia pancreatitis
Source: Cell Death Dis. 2022 Oct 11;13(10):863. doi: 10.1038/s41419-022-05310-w (PMC9554191; doi:10.1038/s41419-022-05310-w)
Supplement: Supplementary file 1 — Supplementary Table S1 [file 41419_2022_5310_MOESM1_ESM.docx]

List of qRT-PCR primers

| Gene name | Primer sequence |
| --- | --- |
| hnRNPA2B1 | Forward: 5’-GCAGGAAGTTCAGAGTTCTAGG  Reverse: 5’-AGTTACTTCCTGGTCCTGGTC |
| HADHA | Forward: 5’-ATGATGGCTTATTACAGTGGCAA  Reverse: 5’-GTCGGAGATTCGTAGCTGGA |
| NEDD8 | Forward: 5’-GAGGCCAAGCCCTGGTATG  Reverse: 5’-CGGGCCGATTGATCTCAGC |
| P65 | Forward: 5’-CCCCACGAGCTTGTAGGAAAG  Reverse: 5’-CCAGGTTCTGGAAACTGTGGAT |
| TRAF2 | Forward: 5’-CCTACTGCTGAGCTCATTCT  Reverse: 5’-CAATCTTGTCCTGGTCTAGC |
| GAPDH | Forward: 5’-GTATGACTCTACCCACGGCAAGT;  Reverse: 5’-TTCCCGTTGATGACCAGCTT |
