# Supplementary figures and images for: Neddylation-mediated degradation of hnRNPA2B1 contributes to hypertriglyceridemia pancreatitis
Source: Cell Death Dis. 2022 Oct 11;13(10):863. doi: 10.1038/s41419-022-05310-w (PMC9554191; doi:10.1038/s41419-022-05310-w)

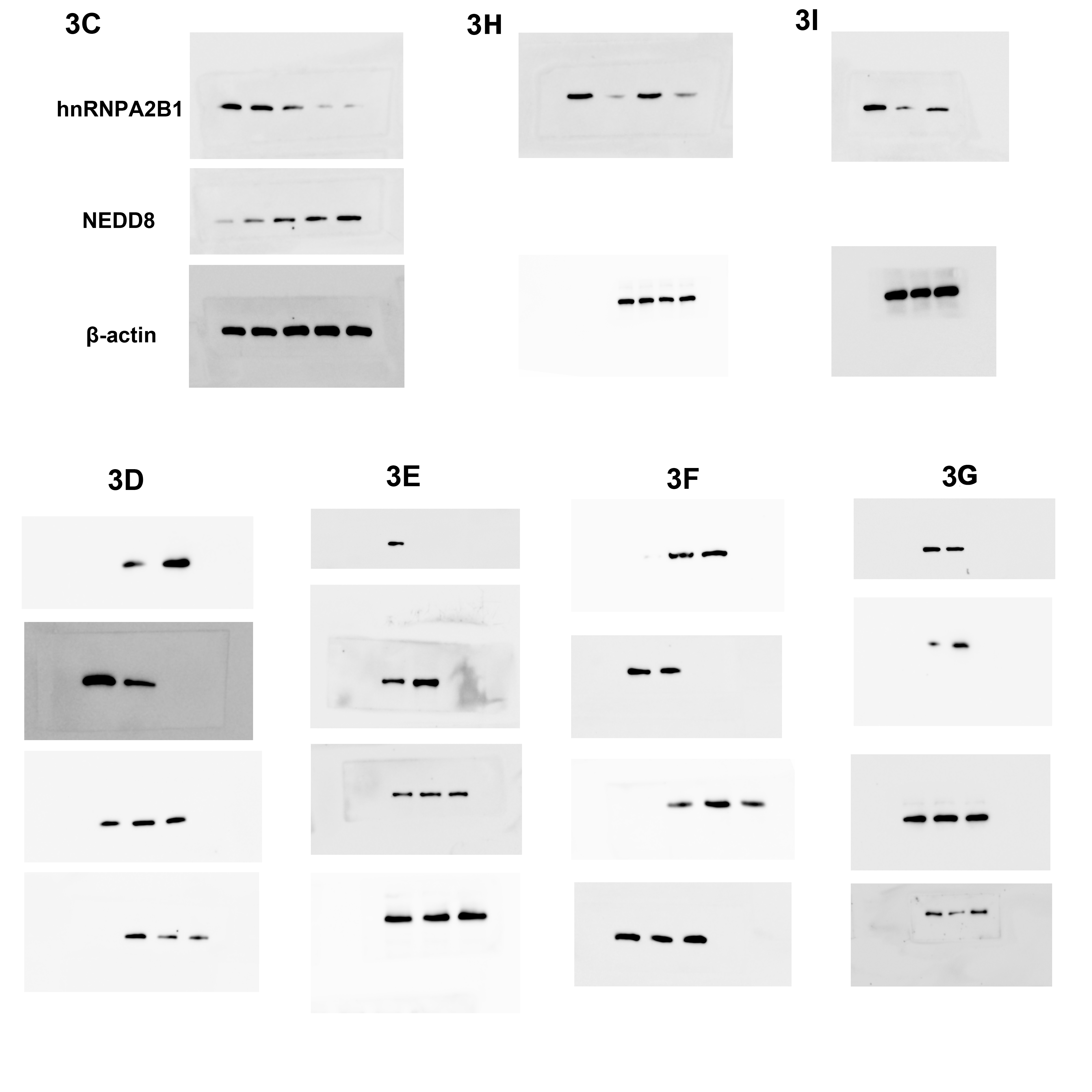

Supplement: Supplementary file 3 — Fig.3-raw blots [file 41419_2022_5310_MOESM3_ESM.tif]

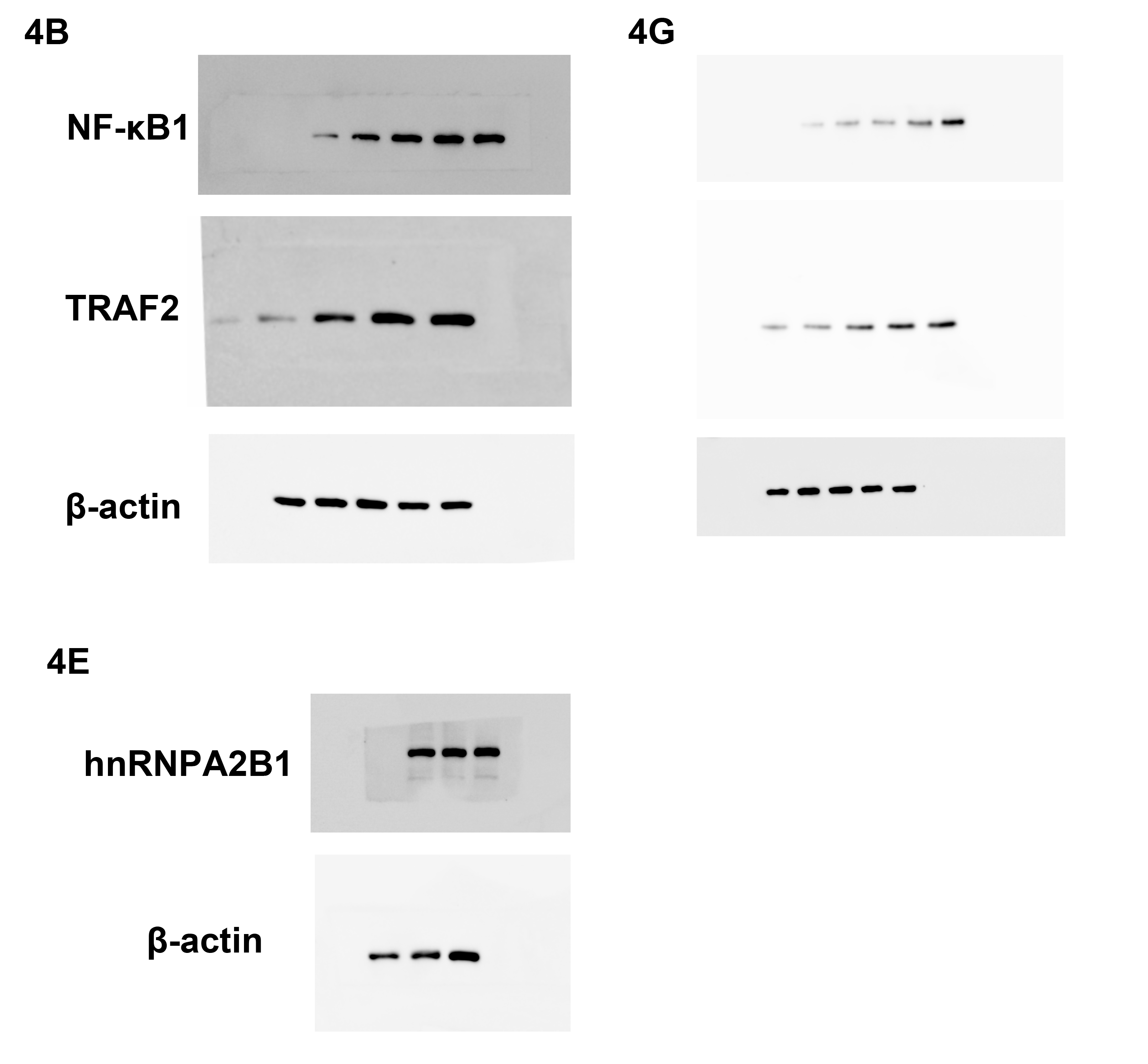

Supplement: Supplementary file 4 — Fig.4-raw blots [file 41419_2022_5310_MOESM4_ESM.tif]

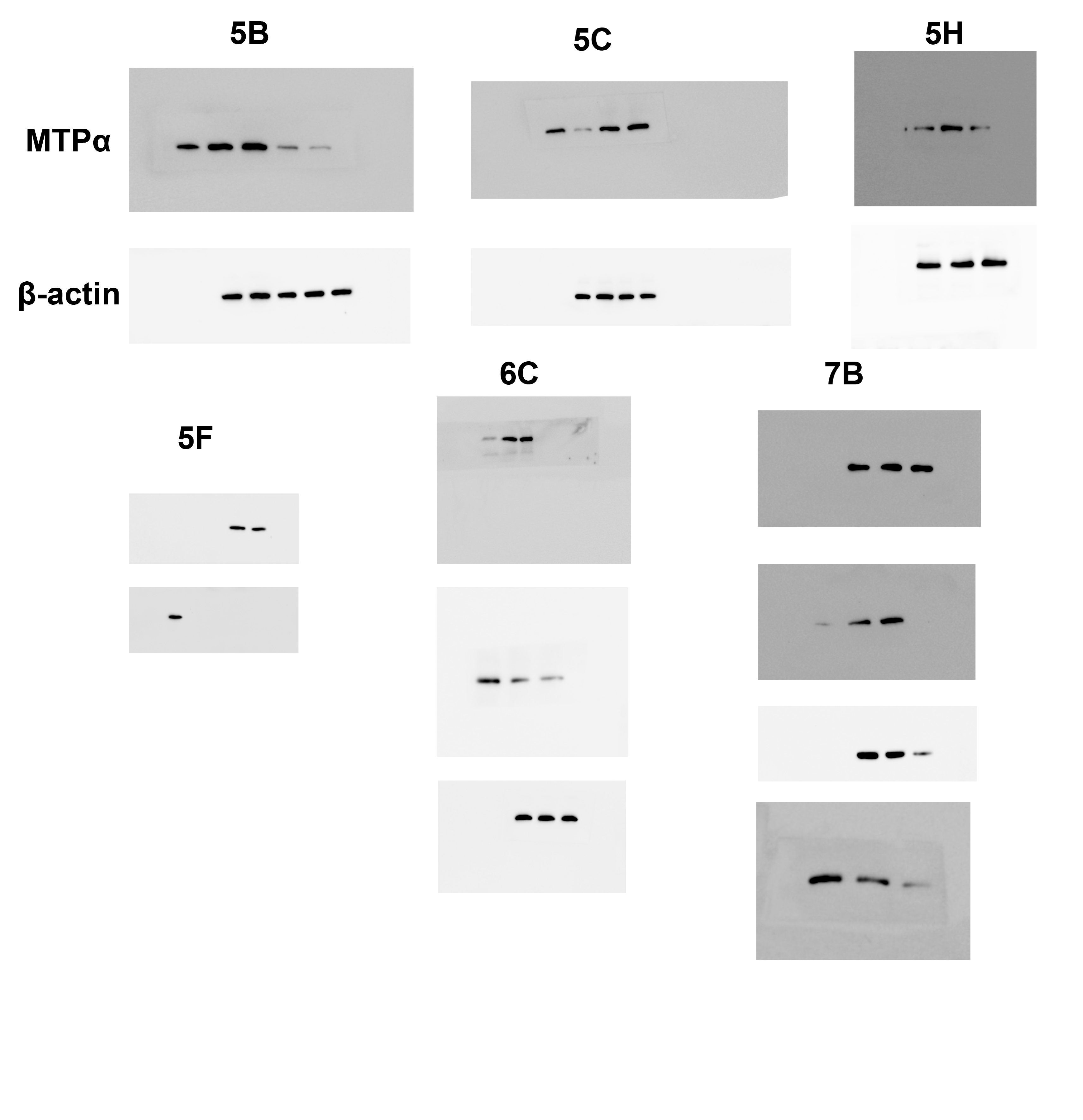

Supplement: Supplementary file 5 — Fig.(5+6+7)-raw blots [file 41419_2022_5310_MOESM5_ESM.tif]

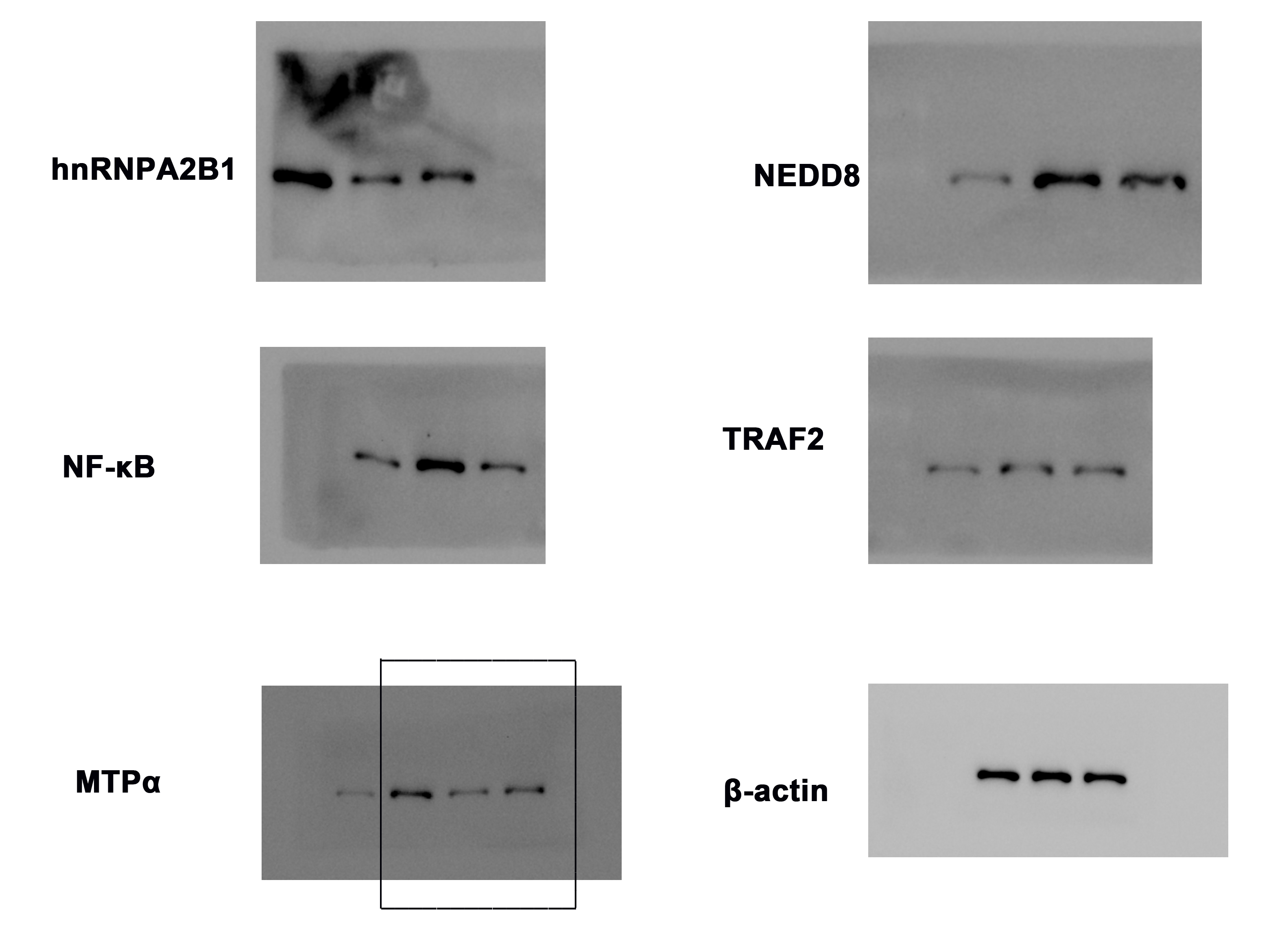

Supplement: Supplementary file 6 — Fig.8-raw blots [file 41419_2022_5310_MOESM6_ESM.tif]
